# CCL2-ethanol interactions and hippocampal synaptic protein expression in a transgenic mouse model

**DOI:** 10.3389/fnint.2014.00029

**Published:** 2014-04-04

**Authors:** Donna L. Gruol, Khanh Vo, Jennifer G. Bray, Amanda J. Roberts

**Affiliations:** Molecular and Cellular Neuroscience Department, The Scripps Research InstituteLa Jolla, CA, USA

**Keywords:** chemokine, neuroimmune, astrocyte, chronic treatment, Western blot, alcohol use disorders

## Abstract

Chronic exposure to ethanol produces a number of detrimental effects on behavior. Neuroadaptive changes in brain structure or function underlie these behavioral effects and may be transient or persistent in nature. Central to the functional changes are alterations in the biology of neuronal and glial cells of the brain. Recent data show that ethanol induces glial cells of the brain to produce elevated levels of neuroimmune factors including CCL2, a key innate immune chemokine. Depending on the conditions of ethanol exposure, the upregulated levels of CCL2 can be transient or persistent and outlast the period of ethanol exposure. Importantly, results indicate that the upregulated levels of CCL2 may lead to CCL2-ethanol interactions that mediate or regulate the effects of ethanol on the brain. Glial cells are in close association with neurons and regulate many neuronal functions. Therefore, effects of ethanol on glial cells may underlie some of the effects of ethanol on neurons. To investigate this possibility, we are studying effects of chronic ethanol on hippocampal synaptic function in a transgenic mouse model that expresses elevated levels of CCL2 in the brain through enhanced glial expression, a situation know to occur in alcoholics. Both CCL2 and ethanol have been reported to alter synaptic function in the hippocampus. In the current study, we determined if interactions are evident between CCL2 and ethanol at the level of hippocampal synaptic proteins. Two ethanol exposure paradigms were used; the first involved ethanol exposure by drinking and the second involved ethanol exposure in a paradigm that combines drinking plus ethanol vapor. The first paradigm does not produce dependence on ethanol, whereas the second paradigm is commonly used to produce ethanol dependence. Results show modest effects of both ethanol exposure paradigms on the level of synaptic proteins in the hippocampus of CCL2 transgenic mice compared with their non-transgenic littermate controls, consistent with ethanol-CCL2 interactions. No evidence of toxic effects of CCL2 or CCL2-ethanol interactions was observed. Taken together, these results support the idea that ethanol induced astrocyte production of CCL2 can result in neuroadaptive changes that interact with the actions of ethanol.

## Introduction

Emerging research indicates that an important effect of alcohol (ethanol) on the brain is the induced production of neuroimmune factors including the chemokine CCL2 (Gruol, 2013). Studies by Qin et al. (2008) showed increased levels of mRNA and protein for CCL2 in the brain of mice subjected to either single or repetitive ethanol exposure (10 days; 5 gm/kg, i.g.). In mice subjected to repetitive ethanol exposure, the increased levels of CCL2 persisted for days after withdrawal from ethanol treatment (Qin et al., 2008), indicating that ethanol can have long-term effects on CCL2 levels. Consistent with these results, Kane et al. (2013a,b) reported that chronic ethanol exposure (6 gm/kg i.g. 10 days) increased levels of CCL2 mRNA in the hippocampus and cerebellum of adult mice. In studies by Whitman et al. (2013), i.g. administration of acute ethanol at a lower dose 2.75 gm/kg did not increase levels of CCL2 mRNA in the brain at either 4 or 24 h after treatment, whereas 24 h after withdrawal from chronic ethanol exposure (liquid diet) levels of CCL2 mRNA were significantly increased in the brain and gradually declined to control levels over the following week. Studies by Flora et al. (2005) showed that i.p. injection of ethanol (2 injections at 3 gm/kg body weight) increased levels of CCL2 mRNA in the hippocampus but not in the corpus striatum, suggesting regional differences in the actions of ethanol on CCL2 production. Importantly, studies of postmortem human brains by He and Crews (2008) showed that specific areas of brains from post-mortem human alcoholics (limbic regions) have a significantly higher concentration of CCL2 than brains from post-mortem human non-alcoholics.

Within the brain, CCL2 is primarily produced by astrocytes and microglia, with upregulated production when the glia are activated (Glabinski et al., 1996). Consistent with this source, exposure of hippocampal-entorhinal cortex brain slice cultures to ethanol (100 mM; 72 h) resulted in a significant and progressive increase (~10 fold increase) in the levels of CCL2 mRNA (Zou and Crews, 2010). Immunostaining of the brain slice cultures showed prominent co-localization of CCL2 immunoreactivity with GFAP immunoreactivity, indicating that astrocytes were the primary source of the ethanol-induced CCL2 production in this preparation (Zou and Crews, 2010). In postmortem brains of human alcoholics, morphological changes in microglia reflective of activation were observed and these changes were associated with upregulation of microglial proteins characteristic of neuroimmune activation (He and Crews, 2008). Microglia activation is a hallmark of neuroimmune activation and is associated with the production of chemokines including CCL2, proinflammatory cytokines and other inflammatory mediators such as COX-2 and iNOS (Blanco and Guerri, 2007; Suk, 2007).

Although research has identified that ethanol induces glial activation and the production of CCL2 in the brain, information on how CCL2 alone or in conjunction with ethanol exposure affects the brain is still relatively limited. Results from several recent studies involving exogenous application of CCL2 and studies in transgenic mice that express elevated levels of CCL2 in the brain indicate that CCL2 can alter neuronal and synaptic function. For example, studies by Zhou et al. (2011) showed that acute exposure to CCL2 (2.5 nM) increased synaptic transmission in hippocampal slices from adolescent mice by acting through presynaptic mechanisms involving transmitter release. Neuronal excitability was also increased by acute exposure to CCL2 in this study. Acute exposure to CCL2 (10 nM) was also reported to increase excitability in striatal neurons, as evidenced by increased spike firing resulting from effects of CCL2 on membrane leak channels (Guyon et al., 2009). In rodent cultured neurons from various brain regions including the cortex, hippocampus, hypothalamus, and mesencephalon, acute exposure to CCL2 elicited Ca^2+^ transients (Banisadr et al., 2005), an effect likely to alter neuronal function. Our studies showed that acute CCL2 (25 nM) decreased excitability and enhanced Ca^2+^ transients elicited by mGluR1 agonists in cultured Purkinje neurons (Van Gassen et al., 2005). CCL2 has been reported to block the activation of T-type Ca^2+^ channels in rodent dorsal root ganglia neurons (You et al., 2010) and to inhibit GABA_A_ receptor-mediated GABAergic responses in cultured spinal cord neurons (Gosselin et al., 2005). Other studies showed neuronal expression and physiological actions of CCL2 in spinal ganglia. For example, Belkouch et al. (2011) showed that acute exposure to CCL2 (100 nM) increased Na^+^ channel activity in primary sensory neurons. In transgenic mice that chronically express high levels of CCL2 (~3000 pg/ml by ELISA) in the hippocampus, CA1 pyramidal neurons showed decreased excitability compared with CA1 pyramidal neurons in non-transgenic littermate controls (Nelson et al., 2011), whereas in another strain of CCL2 transgenic mice that express lower levels of CCL2 (~1500 pg/ml by ELISA) in the hippocampus, CA1 pyramidal neurons showed increased excitability compared with CA1 pyramidal neurons in non-transgenic littermate controls (Bray et al., 2013a), Taken together, these studies support a role for CCL2 as a neuromodulator with actions that vary depending CCL2 concentration and cell type.

In contrast to CCL2, there is an extensive literature documenting the effects of ethanol on the brain. These studies show that a primary mechanism through which ethanol affects the brain is through regulation of neurotransmitter-gated and voltage-gated ion channels that are essential for neuronal excitability and synaptic function, such as the NMDA and GABA_A_ receptors and voltage-gated Ca^2+^ channels (VGCCs) (Crews et al., 1996; Hoffman, 2003). By altering the function of these channels, ethanol alters neuronal properties and ultimately influences the functionality of the brain and consequently behavior. Short-term exposure (hours) to ethanol produces only temporary functional changes. However, repeated (i.e., chronic) ethanol exposure produces persistent neuroadaptive changes that have serious long-term consequences to brain function. For example, chronic exposure to ethanol and/or withdrawal has been reported to increase the density of NMDA receptor binding and elevate the expression of specific NMDA receptor subunits in the brain (Hoffman, 2003). Similar changes in the expression of VGCCs have been reported following chronic alcohol exposure (Dolin et al., 1987). The alterations in NMDA receptors and VGCCs are thought to underlie the hyperexcitability and excitotoxicity associated with withdrawal from chronic alcohol use (Gulya et al., 1991; Whittington et al., 1995).

The action of ethanol to induce the production of CCL2 by brain glial cells and the ability of both ethanol and CCL2 to alter neuronal and synaptic function raises the possibility that the persistently increased levels of CCL2 produced by chronic ethanol exposure could interact with the effects of ethanol on the brain. Several lines of evidence support this possibility. In studies by Breese et al. (2008), rats exposed to CCL2 at weekly intervals (2 injections) by i.c.v. injection prior to chronic ethanol exposure (a 5 day chronic ethanol diet) exhibited reduced social interaction during ethanol withdrawal compared with rats that received the same ethanol treatment but no CCL2. These results indicate that CCL2 can sensitize mice to withdrawal-induced anxiety-like behavior and suggest that CCL2-ethanol interactions can exacerbate the behavioral effects of ethanol. Studies by Blednov et al. (2005) showed that mice lacking CCL2 and/or its receptor CCR2 exhibited lower ethanol preference and consumption than wild-type control mice in a two-bottle choice behavioral paradigm. These results were interpreted as implicating a role for CCL2 in the motivational aspects of ethanol consumption. Our behavioral studies in transgenic mice that persistently express elevated levels of CCL2 in the brain through enhanced astrocyte expression showed that the CCL2 transgenic (CCL2-tg) mice are resistant to the acute ethanol-induced impairments in contextual learning observed in the non-transgenic (non-tg) control mice (Bray et al., 2013b). Consistent with this result, in studies of hippocampal synaptic function *in vitro* acute ethanol (20–60 mM) depressed synaptic plasticity (long-term potentiation; LTP) in the non-tg hippocampus but did not depress LTP the in the hippocampus from CCL2-tg mice (Bray et al., 2013a). LTP is considered to be a cellular mechanism of memory and learning (Lomo, 2003). These *in vitro* results are consistent with an ethanol-CCL2 interaction that involves a protective effect of CCL2 against detrimental effects of acute ethanol on hippocampal synaptic function.

Astrocytes are a primary producer of CCL2 in the brain (Ge et al., 2009). Therefore, targets of ethanol-induced astrocyte produced CCL2 are likely to be the same as astrocyte produced CCL2 in the transgenic mice. This commonality could result in ethanol-CCL2 interactions under conditions of chronic ethanol use when persistently elevated levels of ethanol-induced CCL2 are likely to occur. To address this possibility, we have investigated the effect of exposure to ethanol on the levels of expression of cellular and synaptic proteins in the hippocampus of CCL2-tg and non-tg mice. The elevated levels of CCL2 in the transgenic mice serve as a model for ethanol-induced elevated levels of CCL2, as observed in chronic alcoholics (He and Crews, 2008). Two chronic ethanol exposure paradigms were used, (a) two bottle choice drinking (2BC) and (b) 2BC drinking combined with chronic intermittent ethanol (CIE) exposure (2BC/CIE paradigm). The latter paradigm has been shown to produce ethanol dependence (Finn et al., 2007; Gilpin et al., 2008; Yoneyama et al., 2008; Vendruscolo and Roberts, 2013). CCL2-ethanol interactions were assessed in animals sacrificed approximately 1 month after ethanol exposure, to identify persistent effects of CCL2-ethanol interactions. Results from the Western blot studies are reported here; results from companion electrophysiological and behavioral studies will be reported in a separate publication. The Western blot studies showed modest effects of both ethanol exposure paradigms on the level of synaptic proteins in the hippocampus of CCL2-tg mice compared with their non-tg littermate controls, consistent with ethanol-CCL2 interactions. These results support the proposal of interactions between the effects of CCL2 and ethanol. These interactions do not appear to include toxicity that is persistent in nature, as neither the chronically elevated levels of CCL2 nor CCL2-ethanol interactions produced persistent alterations in several housekeeping proteins in the hippocampus of CCL2-tg mice compared with non-tg mice.

## Materials and methods

### Animals

Mice with astrocyte-targeted overexpression of CCL2 (previously known as monocyte chemoattractant protein-1 or MCP-1) were obtained from Dr. Richard Ransohoff of the Cleveland Clinic Foundation. The generation of the CCL2 transgenic line was described previously (Huang et al., 2002). Briefly, the murine CCL2 gene was placed under control of the huGFAP promoter and purified GFAP-CCL2 fusion gene fragment was injected into fertilized eggs of SWXJ (H-21^q, s^) mice. Transgenic animals and their progeny were identified by analysis of tail DNA. The line was later backcrossed onto a C57BL/6J background and maintained for several years by breeding heterozygous CCL2-tg mice with wildtype C57BL/6J mice. Offspring were genotyped using standard protocols as previously described (Huang et al., 2002). DNA samples were prepared from cut tail tips of individual animals at weaning (21–28 days postnatal) using the Mouse Tail Quick Extraction Kit (Pioneer Inc., San Diego, CA). Mice positive for the human GFAP-murine CCL2 transgene were identified by PCR. Samples were prepared for PCR using the HotStart Taq Master Mix with Loading Dye (Pioneer). All animal procedures were performed in accordance with the Scripps Research Institute and National Institutes of Health Guideline for the Care and Use of Laboratory Animals. Animal facilities and experimental protocols were in accordance with the Association for the Assessment and Accreditation of Laboratory Animal Care.

### Ethanol exposure

Male and female CCL2-tg and non-tg littermate mice at approximately 3–4 months of age were used. The mice were divided into control (i.e., ethanol–naïve) and two ethanol treatment groups. The ethanol-treated mice were exposed to either: (a) two bottle choice ethanol drinking (2BC) or (b) 2BC ethanol drinking combined with CIE vapor exposure (2BC/CIE paradigm). 2BC without vapor does not produce dependence on ethanol, whereas the 2BC/CIE paradigm is commonly used to produce ethanol dependence (Becker and Lopez, 2004; Lopez and Becker, 2005; Finn et al., 2007; Griffin et al., 2009). The experiments involved two different 2BC exposure paradigms (see below). Results from the groups exposed to the two 2BC paradigms were combined for the present analyses and were considered to be ethanol-experienced yet not dependent. The 2BC/CIE paradigm mice were analyzed as a separate group because they had received ethanol vapor in addition to 2BC drinking and had been made ethanol dependent. All studies involved comparisons between CCL2-tg and non-tg mice within a treatment group (i.e., ethanol naïve, 2BC or 2BC/CIE).

#### Two bottle choice ethanol drinking (2BC)

This first 2BC protocol was adapted from that of Blednov et al. (2012). Mice were acclimated for 1 week to individual housing. Two drinking tubes were continuously available to each mouse on Monday through Friday and fluid consumption was measured daily. One bottle of water was available during weekends. Food was available *ad libitum* and mice were weighed every week. After 4 days of water consumption (on Monday, off Friday; water in both tubes), mice were offered 3% ethanol (v/v) vs. water on the following Monday-Friday. Tube positions were changed every day to control for position preferences. Over the following 4 weeks mice received 6, 9, 12, and 15% ethanol in this same manner. Following this, mice received 15% ethanol for 2 h per day for 5 days. The quantity of ethanol consumed (g/kg body weight/24 or 2 h) was calculated for each mouse and averaged across each 4–5 days measurement period. Control animals received water rather than ethanol. Ethanol drinking behavior (preference and consumption) was similar for CCL2-tg and non-tg mice under the conditions examined (choice conditions in non-dependent mice).

#### Chronic intermittent ethanol (CIE) protocol

The specific methodology used is provided on the INIA-West website http://www.scripps.edu/cnad/inia/ under “Methodology,” “SOP Chronic Intermittent Ethanol—Two Bottle Choice Mouse Model PDF” (Becker and Lopez, 2004; Lopez and Becker, 2005; Finn et al., 2007; Griffin et al., 2009). For 15 days (5 days per week for 3 weeks), 30 min before the lights were turned off, mice were singly housed for 2 h with access to two drinking tubes, one containing 15% ethanol and the other containing water (i.e., two bottle choice). Ethanol and water consumption during these 2-h periods was recorded. Following this baseline period, mice were divided, based on equal ethanol and water consumptions in each genotype/sex, into two balanced groups that were exposed to intermittent ethanol vapor (Vapor) or control air (Air) in identical chambers. Following the baseline period, the Vapor group was injected with 1.75 g/kg ethanol plus 68.1 mg/kg pyrazole (an alcohol dehydrogenase inhibitor used to stabilize ethanol levels) and placed in the ethanol chambers to receive intermittent vapor for 3 days (16 h vapor on, 8 h off). Following each 16 h bout of vapor, mice were removed and tail blood was sampled for blood ethanol determination. Target blood ethanol levels were 150–225 mg%. Following the third day of exposure, mice were allowed 72 h of undisturbed time. The Vapor mice were then given 5 days of 2-h access to bottles containing 15% ethanol and water. The Air group (2BC, but no vapor) was injected with 68.1 mg/kg pyrazole in saline and placed in chambers delivering air for the same periods as the Vapor group and were also exposed to two bottle choice drinking at the same time as the Vapor groups. The vapor/air exposure and 5 days of 2 bottle choice testing was repeated another 2 times for a total of 3 rounds of vapor and two bottle choice drinking. Results from the Air group mice were combined with the above-mentioned 2BC mice for data analyses. The 2BC plus vapor group (2BC/CIE) was analyzed separately.

### Protein assays

Protein assays were carried out on lysates prepared from hippocampus of CCL2-tg and non-tg mice. The ethanol-exposed mice were sacrificed approximately 1 month after the termination of ethanol exposure and were approximately 7–9 months of age at the time of sacrifice. To obtain the hippocampi, mice were anesthetized with isoflurane and decapitated. The brains were rapidly removed and immersed in chilled artificial cerebral spinal fluid used for dissections (dACSF). The composition of dACSF was 130.0 mM NaCl, 3.5 mM KCl, 1.25 mM NaH_2_PO_4_, 24.0 mM NaHCO_3_, 0.2 mM CaCl_2_, 5.0 mM MgSO_4_, and 10.0 mM glucose (all chemicals were purchased from Sigma-Aldrich, St. Louis, MO). dACSF was bubbled continuously with 95% O_2_/5% CO_2_ (pH 7.2-7.4). The hippocampus was removed from the brain, snap frozen on dry ice and stored at −80°C until use. Proteins were extracted by sonication in cold lysis buffer containing 50 mM Tris-HCL, pH 7.5, 150 mM NaCl, 2 mM EDTA, 1% Triton X-100, 0.5% NP-40, a Complete Protease Inhibitor Cocktail Tablet (Roche Diagnostics, Mannheim, Germany), and a cocktail of phosphatase inhibitors (Na^+^ pyrophosphate, β-glycerophosphate, NaFl, Na^+^ orthovanadate; all from Sigma-Aldrich). The samples were incubated on ice for 30 min, centrifuged at 13,860 g for 30 min at 4°C, and the supernatants were collected. Protein concentration in the supernatants was determined using the Bio-Rad Protein Assay Kit (Bio-Rad, Hercules, CA). Aliquots were stored at −80°C.

CCL2 levels in protein samples were determined by ELISA using the Mouse CCL2 ELISA Ready-SET-Go! Kit (eBioscience, Inc., San Diego, CA) following manufacturer's instructions. Western blot analysis of protein samples was carried out as previously described (Nelson et al., 2011). Briefly, equal amounts of protein samples were subjected to SDS-PAGE using 4–12% Novex NuPAGE Bis-Tris gels (Invitrogen) and transferred to Immobilon-P membranes (Millipore, Billerica, MA). CCL2-tg and non-tg protein samples from the same treatment group were run on the same gel. Uniform transfer was confirmed by Ponceau S staining (Pierce, Rockford, IL). Membranes were washed and blocked in 5% casein. The membranes were exposed to primary antibodies, washed, and then incubated in secondary antibody coupled to horseradish peroxidase (HRP). Membranes were stripped and reprobed for β-actin. Protein bands were visualized by chemiluminescence and quantified by densitometry measurements using NIH Image software (http://rsb.info.nih.gov/nih-image/). To adjust for possible loading errors, the density of each band was normalized to the density of the band for β-actin in the same lane. Normalized data from CCL2-tg mice were then normalized to values from non-tg mice run on the same gel. Data were combined according to treatment group and reported as mean ± s.e.m. Data reported were obtained from 119 Western blots of hippocampus from 48 transgenic and 55 non-transgenic mice.

The following antibodies were used for Western blot studies: a monoclonal antibody to β-actin (#AC-15, 1:5000; Sigma-Aldrich); a monoclonal antibody to glial fibrillary acidic protein (#MAB360; 1:10,000; Millipore; GFAP); a monoclonal antibody raised against neuron specific enolase (#MAB314; 1:5000; Millipore); a rabbit polyclonal antibody raised against a synthetic peptide to the C-terminus of rat GAD 65/67 (#AB1511; 1:1000; Millipore); a purified rabbit antibody to synapsin 1 (#51-5200; 1:5,000; Invitrogen Life Technologies, Grand Island, NY; Syn 1); a rabbit monoclonal antibody to a synthetic peptide corresponding to amino acids 1–20 at the N-terminus of the mouse vesicular GABA transporter (#NB110-55238; 1-2000; Novus Biologicals, Littleton, CO; VGAT); a rabbit monoclonal antibody to Postsynaptic Density Protein 95 (PSD 95) produced by immunizing rabbits with a KLH-coupled synthetic peptide corresponding to residues surrounding Gly99 of the human PSD95 (#3450; 1:500; Cell Signaling, Danvers, MA); a purified rabbit polyclonal antibody raised against a synthetic peptide of the rat GluA1 subunit of the AMPA receptor conjugated to KLH with a cysteine added (#07-660; 1:500; Millipore); a rabbit monoclonal antibody produced by immunizing rabbits with a synthetic phosphopeptide corresponding to residues surrounding Ser845 of the human GluA1 subunit of the AMPA receptor (#8084; 1-500; Cell Signaling; GluA1p845); a rabbit polyclonal antibody produced by immunizing rabbits with a synthetic short amino acid sequence containing phosphoryated Ser831 of the rat GluA1 subunit of the AMPA receptor (#sc135698; Santa Cruz Biotechnology, Dallas, Texas 75220; GluA1p831); a purified goat polyclonal antibody raised against a peptide corresponding to an amino acid mapping the C-terminus of the human GluN1 subunit of the NMDA receptor (sc-1467; 1:500; Santa Cruz Biotechnology); a purified rabbit polyclonal antibody raised against a synthetic peptide from the 2nd cytoplasmic domain of human GluN2A subunit of the NMDA receptor conjugated to an immunogenic carrier protein (#ab84181;1-500; abcam, Cambridge, MA); a rabbit polyclonal antibody raised against a synthetic peptide mapping to the N-terminus of the human GluN2B subunit of the NMDA receptor (#ab65875; 1-500; abcam); a purified rabbit polyclonal antibody raised against a carboxy terminus peptide of the rat mGluR2/3 conjugated to BSA with gluteraldehyde (#AB1553; 1-1000; Millipore) a rabbit polyclonal antibody raised against p44/p42 mitogen activated protein kinase (#61-7400; 1:5000, Zymed, Carlsbad, CA, USA); a purified rabbit polyclonal antibody raised against a synthetic phospho-peptide (KLH-coupled) corresponding to residues around Thr202/Tyr204 of human p44/p42 MAPK (#9101; 1:500; Technologies, Danvers, AM; pp44/42 MAPK); a purified rabbit polyclonal antibody raised against a synthetic peptide (KLH-coupled) corresponding to the sequence of mouse STAT3 (AB#9132; 1:1000; Cell Signaling Technologies).

### Statistical analysis

Statistical analysis of differences between CCL2-tg and non-tg data was performed using the unpaired *t*-test. Statistical significance was set at *p* < 0.05.

## Results

Neuroadaptive interactions between CCL2 and ethanol in the hippocampus were determined at the level of protein expression by comparing Western blots of CCL2-tg vs. non-tg hippocampus from mice exposed to the same ethanol exposure paradigm. Ethanol naïve mice were also studied. CCL2-tg and non-tg mice were exposed to ethanol by voluntary ethanol drinking using a two bottle choice paradigm (2BC) or a paradigm involving both voluntary ethanol drinking and exposure to ethanol vapor (2BC/CIE).

### Blood ethanol concentration

Because ethanol drinking varied over the exposure period, blood ethanol levels (BEC) were not determined for the 2BC group. BECs during the ethanol vapor exposure bouts did not differ between non-tg and CCL2-tg mice. In non-tg mice average levels were 166 ± 6 mg/dl (*n* = 9) and in CCL2-tg mice average levels were 176 ± 8 mg/dl (*n* = 10).

### CCL2 levels

**Figure 1:**
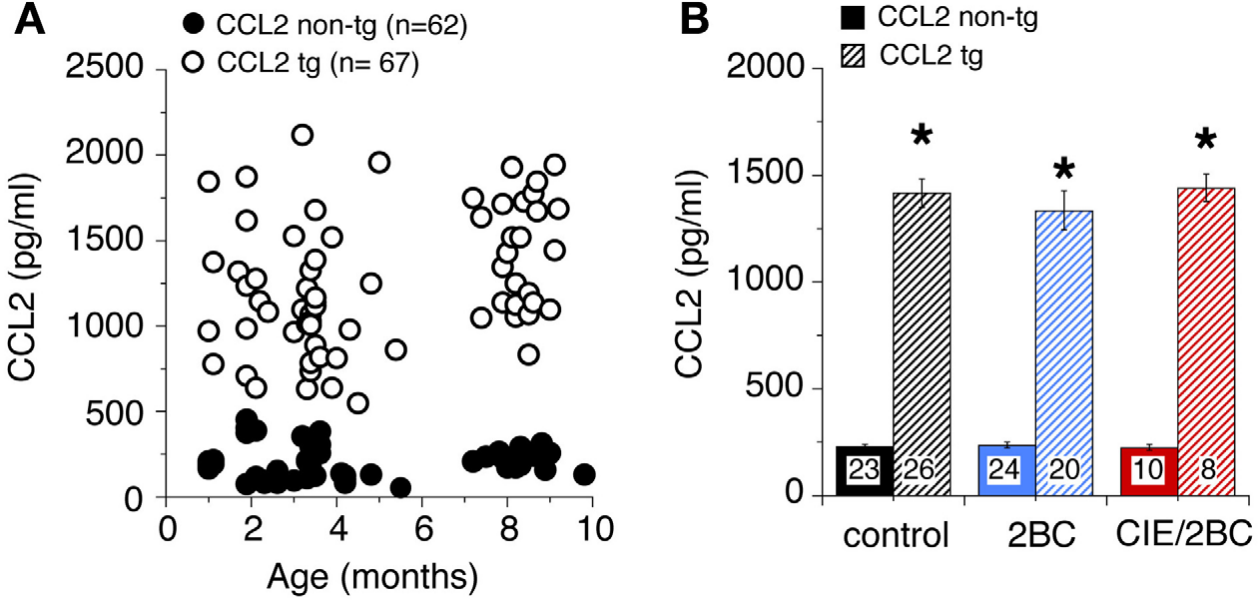
**Levels of CCL2 in CCL2-tg and non-tg hippocampus determined by ELISA. (A)** CCL2 levels in ethanol naïve CCL2-tg and non-tg hippocampus at ages ranging from 1 to 10 months. **(B)** Mean values for CCL2 levels in CCL2-tg and non-tg hippocampus in the three treatment groups. Ethanol naïve and ethanol exposed mice were approximately 7–9 months of age when CCL2 levels were determined (~1 month after the termination of ethanol exposure). Numbers in bars are the number animals measured. ^*^Statistically significant difference (*p* < 0.05, unpaired *t*-test) from non-tg of the same treatment group.

CCL2 levels in the hippocampus of ethanol naïve mice at different ages measured by ELISA are shown in Figure 1A. CCL2 levels were elevated in the hippocampus of CCL2-tg mice at all ages studied (1–10 months of age) and were approximately 6 fold higher in CCL2-tg hippocampus than in non-tg hippocampus. There were no apparent differences in CCL2 levels as a consequence of age (Figure 1A) or ethanol exposure (Figure 1B) for either CCL2-tg or non-tg mice.

### Housekeeping proteins

**Figure 2:**
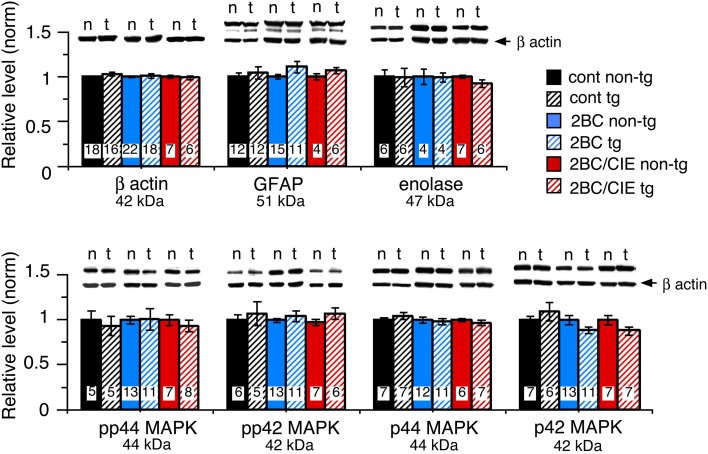
**Levels of housekeeping proteins in CCL2-tg and non-tg hippocampus determined by Western blot.** Values are mean ± SEM. Numbers in bars are the number of animals studied. Representative Western blots are shown above the respective bar. Top blot is the protein indicated for the graph; bottom blot (arrow) is β actin in the same lane. Numbers in bars are the number of animals measured. n, non-tg; t, CCL2-tg.

Several housekeeping proteins were measured to identify potential changes in protein levels that would indicate toxic effects as a result of elevated levels of CCL2 or CCL2-ethanol interactions. Measurements were made of β-actin, a cytosketal protein expressed in all cells, GFAP, an astrocyte specific cytosketal protein, and neuron specific enolase, a specific neuronal protein. No significant difference was observed for the levels of these proteins between CCL2-tg and non-tg hippocampus for ethanol naïve or the ethanol-exposed groups (Figure 2). Levels of p44/42 MAPK, enzymes involved in many cellular processes including synaptic function, were also measured and were similar for CCL2-tg and non-tg hippocampus for ethanol naïve and the ethanol-exposed groups (Figure 2). The level of STAT3, another enzyme involved in many cellular processes, was measured in CCL2-tg and non-tg hippocampus from mice exposed to the 2BC and 2BC/CIE paradigms. Again, there was no significant difference between CCL2-tg and non-tg hippocampus for the level of STAT3 in these two groups (2BC, CCL2-tg = 0.98 ± 0.07, *n* = 3, non-tg = 1.0 ± 0.10, *n* = 4; 2BC/CIE, CCL2-tg = 0.98 ± 0.07, *n* = 10, non-tg = 1.0 ± 0.05, *n* = 4). These results are consistent with a lack of persistent toxicity in the hippocampus as a result of elevated levels of CCL2 or CCL2-ethanol interactions.

### Synaptic proteins

**Figure 3:**
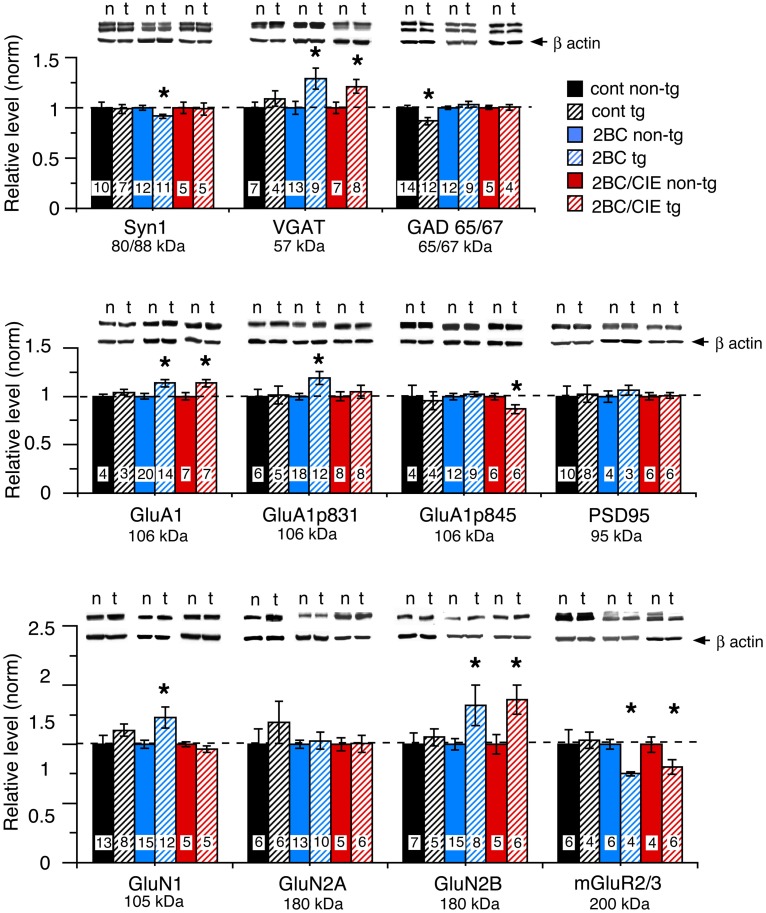
**Levels of synaptic proteins in CCL2-tg and non-tg hippocampus determined by Western blot.** Values are mean ± SEM. Numbers in bars are the number of animals studied. Representative Western blots are shown above the respective bar graph. Top blot is the protein indicated for the graph; bottom blot (arrow) is β actin in the same lane. Numbers in bars are the number of animals measured. n, non-tg, t, CCL2-tg. ^*^statistically significant difference (*p* < 0.05, unpaired *t*-test) from non-tg of the same treatment group.

The level of expression of a variety of hippocampal presynaptic and postsynaptic proteins were examined by Western blot in hippocampus of CCL2-tg and non-tg mice in the ethanol naïve and ethanol-exposed mice. Several differences in the level of pre- and post-synaptic proteins were observed between CCL2-tg and non-tg hippocampus in the ethanol-exposed groups. Only one difference was observed between CCL2-tg and non-tg hippocampus for the ethanol naïve group (Figure 3, Table 1). In general, the differences between CCL2-tg and non-tg hippocampus were modest in nature.

**Table 1:** **CCL2 and CCL2-ethanol interactions affecting hippocampal protein levels**.

**Protein**	Treatment group^*^		
	**Control**	**2BC**	**2BC/CIE**
β-actin	None	None	None
GFAP	None	None	None
Enolase	None	None	None
P44/42 MAPK	None	None	None
Synapsin 1	None	▼	None
VGAT	None	▲	▲
GAD65/67	▼	None	None
PSD95	None	None	None
GluA1	None	▲	▲
GluA1p831	None	▲	None
GluA1 p845	None	None	▼
GluN1	None	▲	None
GluN2A	None	None	None
GluN2B	None	▲	▲
mGluR2/3	None	▼	▼

* For each group, CCL2-tg is compared to non-tg; ▲ = CCL2 tg > non-tg; ▼ = CCL2 tg < non-tg; None, no significant difference between CCL2-tg and non-tg.

Three presynaptic proteins were examined, synapsin 1 (Syn 1), a synaptic vesicle protein, VGAT, the vesicular transporter for the inhibitory transmitter GABA, and GAD65/67, the synthetic enzyme for GABA. The differences were: (a) a significantly lower level of Syn 1 in CCL2-tg hippocampus compared with non-tg hippocampus for the 2BC group, a difference that was not observed in the ethanol naïve or 2BC/CIE groups, (b) a significantly lower level of GAD65/67 in CCL2-tg hippocampus compared with non-tg hippocampus in the ethanol naïve group, a difference that was not observed in the 2BC or 2BC/CIE groups and (c) a significantly higher level of VGAT in CCL2-tg hippocampus compared with non-tg hippocampus for both the 2BC or 2BC/CIE groups, a difference that was not observed in the ethanol naïve group.

The level of several postsynaptic proteins involved in synaptic transmission were also measured including PSD95, a postsynaptic scaffolding protein at excitatory synapses, GluA1, a subunit of the AMPA receptor, GluN1, GluN2A and GluN2B, subunits of the NMDA receptor, and the alpha subunit of Group II metabotropic glutamate receptors (mGluR2/3, the antibody recognizes the alpha subunit of both receptors; the dimer was measured). Two phosphorylated forms of GluA1 that have been shown to play an important role in synaptic plasticity (Sanderson et al., 2008) were also measured, GluA1p831 and GluA1p845 (Soderling and Derkach, 2000; Lee et al., 2010).

Several differences were observed between CCL2-tg and non-tg hippocampus in the 2BC and/or 2BC/CIE groups that were not observed in the ethanol naïve group including (Figures 3B,C): (a) a significantly higher level of GluA1 and GluN2B in CCL2-tg hippocampus compared with non-tg hippocampus in the 2BC group, a difference that was not observed in the ethanol naïve or 2BC/CIE groups, (b) a significantly higher level of GluN1 and GluA1p831 in CCL2-tg hippocampus compared with non-tg hippocampus in the 2BC group, a difference that was not observed in the ethanol naive or 2BC/CIE groups, (c) a significantly lower level of mGluR2/3 in CCL2-tg hippocampus compared with non-tg hippocampus in the 2BC and 2BC/CIE treatment groups, a difference that was not observed in the ethanol naïve group, and (d) a significantly lower level of GluA1p845 in CCL2-tg hippocampus compared with non-tg hippocampus in the 2BC/CIE group, a difference that was not observed in the ethanol naïve or 2BC groups. There was no significant difference in the levels of PSD95 or GluN2A between CCL2-tg and non-tg hippocampus for the ethanol naïve, 2BC or 2BC/CIE groups (Figures 3B,C). Results from the Western blot studies are summarized in Table 1.

## Discussion

In the current study we used a transgenic mouse model that expresses elevated levels of CCL2 in the brain to determine if persistently elevated levels of CCL2 results in neuroadaptive changes that interact with the effects of ethanol exposure. Ethanol is known to induce glial cells of the brain including astrocytes to produce CCL2, an effect that presumably explains the elevated levels of CCL2 in the brains of post-mortem human alcoholics (He and Crews, 2008). Our previous studies using this transgenic model showed interactions between the neuroadaptive effects of CCL2 and the effects of acute ethanol on synaptic plasticity (Bray et al., 2013a). In these studies, the elevated levels of CCL2 protected against the effects of acute ethanol on synaptic plasticity (i.e., LTP).

Studies by others have suggested that ethanol-induced production of neuroimmune factors contributes to the detrimental effects of ethanol on the brain such as toxicity (Crews, 2012). To test for this possibility in the CCL2-tg model we examined the level of several neuronal and glia housekeeping proteins, including β-actin, GFAP, enolase, p42/44 MAPK and STAT3. Levels of these proteins would be expected to be altered as a consequence of cell injury or death. Results showed no difference in the levels of these proteins between CCL2-tg and non-tg hippocampus for either the ethanol naïve or ethanol exposed groups, suggesting that neither CCL2 nor CCL2-ethanol interactions produce persistent toxicity, at least under the conditions of our experiments. However, transient injury could have been produced by ethanol-CCL2 interactions that recovered before our measurements were made at approximately 1 month after termination of ethanol exposure. In our studies, modest levels of ethanol exposure were used (BEC <200 mg/dL), whereas in studies where detrimental effects of CCL2-ethanol interactions were proposed, higher concentrations of ethanol were used. At higher ethanol concentrations CCL2 may act in concert with ethanol or other factors (e.g., glutamate) to produce neurotoxicity or other actions. Thus, the consequences of interactions between CCL2 and ethanol are likely to depend on a number of factors such as ethanol concentration, ethanol exposure paradigm and brain region. The lack of evidence for toxic effects of CCL2 alone observed in this study is consistent with our previous studies of mice at 2–5 months of age. The CCL2-tg mice show little or no brain pathology compared with the non-tg mice as assessed by electrophysiological recordings, Western blot analysis of protein expression and behavioral studies (Bray et al., 2013a).

Because astrocytes and microglia are in close association with synapses (Bessis et al., 2007; Halassa et al., 2007) and dynamically interact with neurons to influence synaptic transmission and plasticity (Ben Achour and Pascual, 2010; Pannasch et al., 2011; Tremblay and Majewska, 2011), ethanol induced glial production of CCL2 could be an important aspect of the effects of ethanol on synaptic function. Our studies of CCL2-tg and non-tg hippocampus in the ethanol naïve group showed that elevated levels of CCL2 alone results in neuroadaptive changes in the level of one important synaptic protein, GAD 65/67. This result is consistent with the ability of astrocyte produced CCL2 to influence synapses, although in this case the change was modest. In contrast, a number of differences in the levels of hippocampal proteins were observed between CCL2-tg and non-tg hippocampus of the ethanol exposed animals, indicating interactions between ethanol and CCL2 that produced neuroadaptive changes. Thus, the levels of VGAT, GluA1 and GluN2B were increased and mGluR2/3 was decreased in CCL2-tg hippocampus compared with the non-tg hippocampus in both the 2BC and 2BC/CIE treatment groups. In addition, levels of GluN1 and GluA1p831 were increased and GluA1p845 and Syn 1 were decreased in CCL2-tg hippocampus compared with non-tg hippocampus in the 2BC treatment group. With the data currently available, it is not possible to determine if similar changes also occurred in non-tg hippocampus of ethanol-exposed mice but to a lesser extent than CCL2-tg hippocampus. Studies in which ethanol naïve and ethanol exposed samples are run on the same Western blot are needed to address this issue, but were not pursued due to limited sample size. In addition, it is unclear why some changes were observed in the 2BC group but not the 2BC/CIE group. It may be that for these proteins both CCL2-tg and non-tg hippocampus were similarly affected by the 2BC/CIE exposure paradigm and thus changes would not be evident when comparisons were made between CCL2-tg and non-tg hippocampus. Alternatively, changes produced by 2BC/CIE treatment were transient and not evident at the post ethanol exposure time period examined in this study. Future studies will be needed to address these possibilities. Future studies will also be required to identify the mechanisms underlying the interactions between ethanol and CCL2. This interaction is likely to involve alterations in the expression of target receptors for CCL2 or the activation of downstream signaling pathways. However, taken together, results from the 2BC studies indicate that even moderate levels of prolonged ethanol exposure can interact with the effects of persistently elevated levels of CCL2 to cause neuroadaptive changes.

Results from the current studies are consistent with previous studies showing the ability of chronic ethanol exposure paradigms to result in increased levels of synaptic proteins. For example, GluA1 is increased by chronic ethanol exposure in rat cortical cultures (Chandler et al., 1999) and other preparations (Holmes et al., 2013). Similarly, a number of studies have shown that chronic ethanol exposure and withdrawal results in elevated levels of NMDA receptor subunits and synaptic responses in the hippocampus and other brain regions (Nelson et al., 2005; Qiang et al., 2007; Nagy, 2008). VGAT has also been reported to be increased by chronic ethanol exposure in studies involving a culture preparation of cortical neurons (Zink and Spanagel, 2005). However, the changes in the level of synaptic proteins produced by ethanol exposure similar to that used in our studies are typically transient in nature (Roberto et al., 2006; Clapp et al., 2010; Pian et al., 2010), whereas the interactions between CCL2-ethanol observed in our study were evident 1 month following termination of ethanol exposure. These results suggest that CCL2-ethanol interactions can produce changes similar to that of ethanol alone but that the changes are more persistent in nature.

Several studies have shown that ethanol exposure increases the levels of CCL2 in the brain (Flora et al., 2005; He and Crews, 2008; Qin et al., 2008; Kane et al., 2013a). Measurement of CCL2 levels in the hippocampus of control mice by ELISA showed approximately a 6 fold higher concentration of CCL2 in CCL2-tg hippocampus compared with non-tg hippocampus at all ages studied. However, alterations in the levels of CCL2 levels by ethanol treatment were not evident for either the non-tg or CCL2-tg hippocampus. These results suggest that our ethanol exposure paradigm does not result in persistently elevated levels of CCL2, presumably due to the modest ethanol BECs achieved. However, information on trafficking of CCL2 in the brain is limited and the rate of production and relative level of stored vs. secreted CCL2 are unknown and could be affected by ethanol exposure in a manner not detectable by our ELISA measurements. Because ethanol was not on board when measurements of protein levels were made, the ethanol-CCL2 interactions observed in our studies are considered to reflect persistent neuroadaptive changes that once established do not require the presence of ethanol for continued expression, at least for a 1 month period following withdrawal from ethanol.

Expression of CCL2 in the transgenic mice is under control of the GFAP promoter. Astrocytes start to produce GFAP about 1 day postnatal and expression continues throughout the lifetime of the animal (Kim et al., 2011). Our measurements of CCL2 levels in CCL2-tg mice showed elevated levels at the earliest ages measured, 1 month postnatal. Thus, results from the CCL2-tg model are likely to be most relevant to effects of chronic ethanol use that starts early in the lifespan, such as the juvenile or adolescent stage of life, a pattern of ethanol use that has significant risk for developing ethanol dependence and is currently an important societal issue (Hingson et al., 2006; Foltran et al., 2011).

## Author contributions

Donna L. Gruol, Amanda J. Roberts, and Jennifer G. Bray designed the studies and wrote the paper. Donna L. Gruol, Jennifer G. Bray, and Khanh Vo managed the mouse colony. Jennifer G. Bray performed the dissections. Donna L. Gruol, and Khanh Vo carried out the biochemical analyses.

### Conflict of interest statement

The authors declare that the research was conducted in the absence of any commercial or financial relationships that could be construed as a potential conflict of interest.
